# Knowledge, attitudes, and practices of ICU nurses regarding daily sedation interruption: a cross-sectional study

**DOI:** 10.3389/fmed.2026.1822534

**Published:** 2026-05-20

**Authors:** Lixia Hu, Xiaoli Du, Pingwen Zhang, Yan Luo, Xiuru Yang, Fenglin Yan, Dan Wen, Feifei Xie

**Affiliations:** Intensive Care Unit, School of Medicine, Mianyang Central Hospital, University of Electronic Science and Technology of China, Mianyang, China

**Keywords:** attitudes, critical care, daily sedation interruption, ICU nurses, knowledge, practice

## Abstract

**Background:**

Appropriate analgesia and sedation are essential in intensive care units (ICU). Daily sedation interruption (DSI) minimizes sedation and maximizes patient care by interrupting or reducing sedative infusion.

**Objective:**

This study aimed to assess the knowledge, attitudes, and practices of intensive care unit nurses regarding daily sedation interruption and to identify factors associated with its implementation in clinical practice.

**Methods:**

A cross-sectional survey was conducted among ICU nurses from five general hospitals in Sichuan Province, China, from July 29, 2024, to August 20, 2024, using convenience sampling. The nurses completed a self-designed online questionnaire on the DSI that included three dimensions: knowledge, attitude, and practical behavior. This study followed the STROBE (Strengthening the Reporting of Observational Studies in Epidemiology) guidelines for reporting cross-sectional studies.

**Results:**

In total, 343 valid questionnaires were collected, with a recovery rate of 95.3%. The DSI knowledge score of ICU nurses was 4 (2–4) points, with a scoring rate of 40%. The DSI attitude score of ICU nurses was 37 (31–40) points, with a scoring rate of 92.5%. Forty-two percent (144) of nurses had received DSI training, and 2.6% (9) of nurses had never implemented DSI. The most commonly used sedation assessment tool was the Richmond Agitation Sedation Scale (RASS) (80.8%). The most common impediment to DSI implementation was increased incidence of self-removal of tracheal tubes and other catheters (71.4%). There were significant differences in DSI knowledge among nurses by age, gender, professional title, education level, and years working in the ICU (*p* < 0.05). There were statistically significant differences in the attitudes of ICU nurses toward DSI by age, professional title, educational level, and years of work in the ICU (*p* < 0.05).

**Conclusion:**

DSI knowledge among ICU nurses is insufficient, recognition is high, and practice behaviors need to be strengthened. ICU nurses need more training and guidance in the knowledge and practice of DSI.

## Background

Critically ill patients often suffer from pain and anxiety caused by various types of intubation, mechanical ventilation, and trauma (1). Anxiety and agitation were reported in more than 70% of critically ill patients (2). These patients frequently exhibit widespread tension, fear, loneliness, insomnia, and irritability. Such psychological impacts can interfere with clinical monitoring and treatment, exacerbate the patient’s stress response, increase bodily oxygen consumption, and add to the burden on vital organs (3). For critically ill patients, maintaining a low metabolic state can alleviate various stress responses and inflammatory damage, and reduce organ damage. The concept of moderation has been emphasized in the sedation of ICU patients. Excessive or insufficient sedation may negatively affect the prognosis of the patients (4). Kress et al. (5) noted that oversedation can obscure the distinction between drug effects and neurological deterioration, impeding accurate neurological assessment. Furthermore, continuous infusion of sedatives has been identified as an independent predictor of prolonged mechanical ventilation and extended intensive care units (ICU) stay (6).

Daily sedation interruption (DSI) was first proposed by Kress in 2000 (5). It involves temporarily stopping or reducing sedative infusions daily until the patient is awake and can follow simple commands. DSI is included among the recommended measures for managing mechanically ventilated patients in U. S. analgesia and sedation guidelines (7). Numerous studies have demonstrated that DSI can effectively reduce the duration of mechanical ventilation, ICU length of stay, and overall hospital stay (8). However, its implementation in clinical practice is not optimal. A multicenter study from Australia and New Zealand showed that up to 68% of patients were deeply sedated within the first 48 h of mechanical ventilation (9). A survey of sedation practices in Northern Europe found that DSI was used in only 15% of ICUs (10). The successful implementation of DSI and sedation protocols in the ICU mainly relies on nurses. They are responsible for continuous monitoring, assessment, and adherence to the protocols. A study on DSI among pediatric ICU nurses in China indicated that fewer than 50% of nurses implemented DSI, and their knowledge regarding DSI was insufficient (11). However, The knowledge and practice of DSI among adult ICU nurses in China remain unclear. Therefore, we conducted this cross-sectional study to investigate the knowledge, attitudes, and practices of ICU nurses in China regarding DSI.

## Methods

### Study design

A cross-sectional survey was conducted from July 29, 2024, to August 20, 2024.

### Setting

The study was conducted in the intensive care units of five tertiary general hospitals in Sichuan Province, China. The selection criteria for the hospitals are based on being comprehensive hospitals. The hospitals are located in different cities (Mianyang, Chengdu, Deyang, Suining, and Guangyuan) to represent varied regional practices within the province. The nurses worked in general, pediatric, cardiac, respiratory, and neurosurgical ICUs.

### Participants

A convenience sampling method was used to recruit participants from the five participating hospitals. The inclusion criteria were registered nurses, ICU clinical work time ≥1 year, and voluntary participation in the survey. Interns, training nurses, and nurses who were absent during the survey period (e.g., sick leave and maternity leave), were excluded. The study protocol was approved by the Ethics Committee of the Mianyang Central Hospital (approval number: S20240233-02). Based on Kendall’s sample size estimation principle, the sample size is 5–10 times the number of items. The survey questionnaire includes 23 items, and considering a 20% invalid response rate, the estimated sample size is 138 to 276 cases.

### Questionnaires

#### Demographic profile

The research team designed a demographic questionnaire that included information on sex, age, education, professional title, and years of experience in the ICU.

#### DSI knowledge, attitudes, and practices

Adapted from Hongqin Zhou, the questionnaire was originally used to investigate the knowledge, attitudes, and practices of pediatric nurses regarding DSI (11). Permission was obtained from the authors to use the questionnaire before conducting the survey. To ensure its validity and relevance for adult ICU nurses, the adaptation process included expert consultation. A panel of seven experts (two ICU physicians, three ICU nurse managers, and two graduate nursing students) evaluated the initial adapted version for content relevance, clarity, and comprehensiveness. Their feedback was used to refine items, update clinical scenarios for the adult ICU context, and ensure alignment with current guidelines. The revised questionnaire was then pre-tested with 10 ICU nurses to further assess clarity and feasibility, leading to minor wording adjustments. The nurses who participated in this pilot test were not included in the main study sample. The content validity of the final questionnaire was formally evaluated by the same expert panel. Experts rated each item for relevance on a 4-point scale. We calculated both the Item-level Content Validity Index (I-CVI) and the Scale-level CVI using the Average method (S-CVI/Ave). The S-CVI/Ave for the entire scale was 0.871. The internal consistency reliability of the questionnaire was assessed using Cronbach’s alpha. The overall alpha was 0.923. The domain-specific alphas were 0.892 for the Knowledge dimension and 0.916 for the Attitude dimension.

The knowledge dimension includes the DSI concept, indications, applications, precautions, and drug administration methods. Each entry was awarded two points for correct answers and zero points for incorrect answers, resulting in a range of 0–10 points. The total knowledge score ranged from 0 to 10 points. A scoring rate was calculated as (Total Knowledge Score/10) * 100% to reflect the percentage of the total possible score achieved.

The attitude dimension included nurses’ recognition of DSI and their willingness to implement DSI. Recognition of DSI includes eight items that can shorten ventilation time and hospital stay, reduce infections and complications, reduce drugs and costs, reduce psychological burden, and prevent post-traumatic stress syndrome. A five-point Likert scale was used for scoring. Scores 1–5 corresponded to the following answers: “completely disagree,” “disagree,” “neutral,” “agree,” and “fully agree.” The total score ranged from 8 to 40 points, and the higher the score, the higher the recognition. The scoring rate was calculated by dividing the actual score by the total score. The willingness to implement DSI includes two options: “Yes” and “no.”

The practical dimensions included whether the nurses had received DSI training, frequency of DSI implementation, whether the department had sedation programs, frequency of sedation scoring, sedation scoring tools, intravenous sedation methods, analgesic use, common problems with increasing sedation, and obstacles to DSI implementation. Each entry was selected once or multiple times according to the given options. Frequency was defined as never = 0 times/week, rarely = 1–2 times/week, often = 3–5 times/week, always 6–7 times/week. These projects were subjected to descriptive analysis as independent variables.

#### Data collection and quality control methods

The Wenjuanxing platform was used to distribute and retrieve the electronic questionnaires.^1^ Before initiating the study, the research team contacted the ICU head nurse at the hospital where the survey was conducted. Participants were informed of the purpose and content of the survey, and the link and QR codes of the questionnaire were released after obtaining their consent. The questionnaire used a unified guide to explain the purpose, content, and precautions of the study. Electronic informed consent was obtained from all participants prior to their access to the online survey. Questionnaires that took less than 120 s to complete were considered invalid and excluded from analysis. All responses were screened for logical inconsistencies. The same IP address could only be entered once.

### Statistical analysis

SPSS 26.0 (IBM, New York, NY, United States) statistical software was used for data analysis. Categorical variables were described using frequencies and percentages. The normality of continuous data was assessed using the Kolmogorov–Smirnov test, supplemented by visual inspection of Q-Q plots and histograms. Continuous variables were expressed as mean ± standard deviation when they conformed to a normal distribution, and as median and quartile when they did not conform to normal distribution. Independent samples t-tests or one-way ANOVA were used to compare normally distributed knowledge and attitude scores across different groups. The Mann–Whitney U or Kruskal-Wallis H tests were applied for non-normally distributed scores. The Chi-square test was used to compare categorical variables between groups. The statistical test level was *α* = 0.05, where a *p* < 0.05 indicated statistical significance.

## Results

### Demographic characteristics

A total of 360 questionnaires were distributed and 343 were recovered, with a valid recovery rate of 95.3%. A total of 174 respondents (50.7%) were aged between 20 and 30. There were 53 (15.5%) respondents had specialist degree, 280 (81.6%) had bachelor’s degree, and 10 (2.9%) had master’s degree or higher. The detailed demographic characteristics of the participants are summarized in Table 1.

**Table 1:** Comparison of knowledge and attitude toward DSI among ICU nurses with different characteristics (*n* = 343).

Characteristic	Number (%)	Knowledge median (interquartile range)	*Z*	*p*	Attitude median (interquartile range)	*Z*	*p*
Age			14.559	0.002		18.054	<0.001
20–30 years	174(50.7%)	4(2–4)			32(29–40)		
31–40 years	141(41.1%)	4(2–4)			40(32–40)		
41–50 years	25(7.3%)	4(2–6)			33(30.5–40)		
≥51 years	3(0.9%)	6(6–6)			34(28–40)		
Gender			−3.808	<0.001		−1.076	0.282
Female	265(77.3%)	4(2–4)			37(30.5–40)		
Male	78(22.7%)	4(4–6)			37(31.75–40)		
Years of working in ICU			22.001	<0.001		8.547	0.036
≤5 years	172(50.2%)	2(2–4)			36.5(29–40)		
6–10 years	44(12.8%)	4(2–4)			38(35–40)		
11–15 years	121(35.3%)	4(4–6)			37(32–40)		
≥16 years	6(1.7%)	5(2–6)			28(28–36.25)		
Professional title			6.447	0.040		16.283	<0.001
Junior professional titles	159(46.4%)	4(2–4)			32(29–40)		
Intermediate professional titles	173(50.4%)	4(2–4)			40(32–40)		
Senior professional titles	11(3.2%)	4(2–6)			32(30–40)		
Education			8.633	0.013		19.504	<0.001
Specialty degree	53(15.5%)	4(4–5)			32(29–36)		
Bachelor’s degree	280(81.6%)	4(2–4)			39(32–40)		
Master’s degree and above	10(2.9%)	4(2–6)			32(28–37)		

### Knowledge scores of ICU nurses on DSI

The DSI knowledge score of ICU nurses was 4 (2–4) points, with a score rate of 40%. A total of 24 (7.0%) had the lowest score of 0, and three (0.9%) had the highest score of 10. The correct rates for the DSI concept was 32.9%, the correct rate for application scenarios was 56.3%, the correct rate for precautions was 29.2%, the correct rate for drug administration methods was 35.9%, and the correct rate for indications was 19.2%.

### Attitude scores of ICU nurses on DSI

The ICU nurses’ attitude toward DSI was 37 (31–40) points, with a scoring rate of 92.5%. A total of 16 (4.7%) had the lowest score of 9 and 142 (41.4%) had the highest score of 40. The scores for each item are listed in Table 2. A total of 266 nurses (77.6%) were willing to implement DSI, whereas 77 nurses (22.4%) were not.

**Table 2:** ICU nurses’ attitude scores toward DSI ( $\bar{x}$ ± s, *n* = 343).

Item	Score
1. DSI can reduce the time of mechanical ventilation, ICU stay and total hospital stay.	4.32 ± 0.91
2. DSI can reduce the incidence of nosocomial infection.	4.14 ± 1.22
3. DSI can reduce the complications of mechanical ventilation and the rate of tracheotomy.	4.28 ± 1.07
4. DSI can reduce additional neurological tests (such as CT, MRI, lumbar puncture, etc.).	4.05 ± 1.28
5. DSI can reduce the amount of sedative and analgesic drugs.	4.19 ± 1.17
6. DSI can reduce the total cost of hospitalization.	4.18 ± 1.17
7. DSI can reduce the occurrence of negative psychological events in patients.	4.27 ± 0.99
8. DSI can reduce the incidence and symptoms of post-traumatic stress syndrome.	4.30 ± 0.98

### DSI practice behavior of ICU nurses

42.0% (144) nurses had received DSI training. 2.6% (9) nurses had never implemented DSI, and 52.5% (180) nurses indicated that they rarely implemented DSI. 84.5% (290) nurses indicated that the department had a sedation program. 28.3% (97) nurses evaluated or scored sedation every 2 h. The most commonly used sedation assessment tool was the Richmond Agitation Sedation Scale (RASS) (80.8%) 0.80.5% (276) nurses administered sedative drugs by continuous intravenous infusion, with bolus injection if needed. 6.1% (21) sedation was always accompanied by analgesic treatment. Reasons for increased sedation included man–machine confrontation (46.6%), patient agitation/confusion/delirium (71.7%), patient self-removal of drainage tube/infusion tube/catheter (65.0%), patient discomfort or pain (43.1%), and increased nurse workload (38.5%). The top three hindrance factors for DSI implementation were increased incidence of patients’ self-removal of tracheal tube or other catheters (71.4%), agitation and discomfort (70.8%), and man–machine confrontation (49.9%) (see Table 3).

**Table 3:** DSI practice behavior of ICU nurses (*n* = 343).

Item	Number (percentage)
Have you received DSI training?	
Yes	144(42.0%)
No	199(58.0%)
Frequency of DSI implementation never	
Never	9(2.6%)
Rarely	180(52.5%)
Often	122(35.6%)
Always	32(9.3%)
Does the department have a sedation program?	
Yes	290(84.5%)
No	53(15.5%)
Sedation assessment frequency	
Never	41(12.0%)
1 h	67(19.5%)
2 h	97(28.3%)
4 h	67(19.5%)
6 h	71(20.7%)
Sedation assessment tool	
Bispectral index, BIS	5(1.4%)
Sedation-agitation scale, SAS	21(6.1%)
Richmond agitation and sedation scale, RASS	277(80.8%)
Ramsay sedation scale, RSS	40(11.7%)
Intravenous sedation methods	
Continuous intravenous infusion	47(13.7%)
Intermittent intravenous infusion	20(5.8%)
Continuous intravenous infusion, rapid infusion if needed	276(80.5%)
Sedation with additional analgesics	
Never	62(18.1%)
Rarely	233(67.9%)
Often	27(7.9%)
Always	21(6.1%)
Reasons for increased sedation	
Man–machine confrontation	160(46.6%)
Patient agitation/confusion/delirium	246(71.7%)
Patient self-removal of drainage tube/infusion tube/catheter	223(65.0%)
Patient discomfort or pain	148(43.1%)
Increased nurse workload	132(38.5%)
Hindrance factors for DSI implementation	
Cause agitation and discomfort in patients	243(70.8%)
Cause man–machine confrontation	171(49.9%)
Increased incidence of patients’ self-removal of tracheal intubation or other catheters	245(71.4%)
Increase nurse workload	139(40.5%)
Nurses lack knowledge of DSI	151(44.0%)

### Comparison of knowledge and attitude toward DSI among ICU nurses with different characteristics

General demographic information was used as the independent variable to analyze the differences in knowledge and attitudes regarding DSI among nurses with different characteristics. The results of the univariate analysis showed statistically significant differences in DSI knowledge among ICU nurses of different ages, genders, professional titles, educational levels, and years of working in the ICU (*p* < 0.05). There were statistically significant differences in the attitudes of ICU nurses toward DSI according to age, professional title, educational level, and years of work in the ICU (*p* < 0.05). Further details are presented in Table 1.

## Discussion

The DSI knowledge score of ICU nurses in China was 4 (2–4) points, with a score rate of 40%, which was at a low level. There were significant differences in DSI knowledge among nurses of different ages, sexes, professional titles, education levels, and years working in the ICU (*p* < 0.05). ICU nurses had the weakest knowledge of DSI indications, with only 19.2% accuracy. Most nurses have difficulty determining which patients are suitable for DSI, which represents a significant deviation from current international guidelines. The 2018 SCCM PADIS guidelines recommend considering DSI for adults receiving mechanical ventilation, highlighting the need for protocolized patient selection (12). The management guidelines revised in Germany in 2015 also emphasize that DSI should be performed in ICU patients with deep sedation with RASS score ≤ − 2 without conjunctures (13). We also found deficiencies in nurses’ knowledge of DSI concepts, precautions, drug administration methods, and application contexts. The correct rate for DSI concept was 32.9%, indicating that nurses had not formed a clear understanding of the basic concepts of DSI. The correct rate of drug administration was 35.9%, indicating that nurses also had some cognitive defects in drug administration. The precautions were correct 29.2 percent of the time, indicating that nurses may have overlooked complications such as accidental extubation when implementing the DSI, thereby increasing perceived and real risks, which in turn becomes a barrier to practice. It is worth noting that the correct rate of application situation was 56.3%, which is relatively high. However, this still indicates that nearly half of the nurses lack accurate judgment on the application of DSI in different clinical situations, which also requires attention. This knowledge deficit directly translates to hesitation in practice and may contribute to adverse patient events, undermining the potential benefits of DSI.

The ICU nurses’ attitudes toward DSI scored 37 (31–40) points, with a scoring rate of 92.5%, which was at a high level. This indicates that ICU nurses have a positive attitude toward the clinical significance of DSI. However, this presents a critical disconnect between belief and implementation. There were statistically significant differences in the attitudes of ICU nurses toward DSI according to age, professional title, educational level, and years of work in the ICU (*p* < 0.05). The items with high acceptance were that DSI reduced the duration of mechanical ventilation, length of stay in ICU, and total length of stay (score 4.32 ± 0.91), reduced the incidence of post-traumatic stress syndrome, reduced related symptoms (score 4.30 ± 0.98), and reduced the rate of mechanical ventilation complications and tracheotomy (score 4.28 ± 1.07). A meta-analysis found that DSI is an effective strategy for reducing the duration of mechanical ventilation in critically ill patients, particularly those with higher disease severity (14). They also found that DSI significantly reduced ICU stay time, sedation time, tracheotomy risk, and ventilator-associated pneumonia risk. Although ICU nurses held a positive attitude toward DSI, 22.4% of nurses were still unwilling to implement DSI in clinical practice. This mismatch between high attitudinal scores and persistent reluctance in a significant minority underscores that knowledge deficits and practical barriers, rather than a lack of belief in its benefit, are the primary obstacles to changing practice. The failure to bridge this attitude-practice gap has a direct, negative impact on patient outcomes by perpetuating deep sedation practices associated with longer ventilation times and hospital stays. Concerns regarding respiratory disorders and the risk of unplanned extubation are important factors hindering medical staff from implementing DSI (15). Tanios et al. believed that the limited acceptance of nurses is one of the factors hindering the clinical application of DSI (16). Studies have shown that the use of DSI in patients on mechanical ventilation does not increase the incidence of adverse events or complications (17, 18). However, limited knowledge of DSI has weakened the enthusiasm of medical staff for applying DSI. In addition, DSI may increase the workload of medical staff, which hinders its clinical application (19).

The majority (58.0%) of ICU nurses did not receive DSI training. This fundamental training gap is a key system-level issue underlying many of the observed practice deficiencies. The frequency of DSI implementation was not high, with 55.1% of nurses reporting that they never or rarely performed DSI, similar to Borkowska’s study (20). The direct impact of this low implementation rate is the forfeiture of documented patient benefits, including reduced duration of mechanical ventilation and ICU length of stay (8). This finding highlights a gap in implementing the “Awakening and Breathing Coordination” component of the ABCDEF bundle, an integrated care approach designed to improve outcomes in mechanically ventilated patients. The American Society of Critical Care Medicine points out that the RASS and SAS are the most reliable assessment tools for measuring sedation quality and depth in adult ICU (12). In our study, 80.85% of nurses chose RASS for sedation assessment, which was slightly lower than Morandi et al. 90–93% scale use frequency (21). Moreover, the selection of the assessment tools in our study was not uniform and lacked pertinence. The guidelines recommend sedation evaluations every 46 h, while avoiding evaluations during sleep (12). The frequency of evaluation should be adjusted when clinical interventions are likely to cause pain. The majority of nurses in our study met the guidelines for sedation assessment. For intravenous sedation, 80.5% of nurses chose “continuous infusion, rapid infusion if needed”, which was contrary to the goal of DSI. This preference for continuous infusion directly conflicts with the pharmacokinetic rationale of DSI and sustains a state of deeper sedation than necessary, potentially prolonging ventilator dependence. In our study, only 6.1% of nurses used analgesia along with sedation; and the most common reasons for increased sedation were agitation, confusion, and delirium (71.7%). It has been suggested that analgesia should be administered simultaneously or before sedation (22). This practice critically deviates from the “analgesia-first” principle that is central to contemporary light sedation strategies, such as the eCASH concept, and the PADIS guidelines, which emphasize treating pain to minimize sedative needs. The primary causes of irritability in ICU patients are pain and discomfort. Analgesia should be used as the basis for sedation (23). The top three barriers to DSI implementation were increased incidence of spontaneous removal of tracheal tubes or other catheters (71.4%), agitation and discomfort (70.8%), and human-machine confrontation (49.9%), which were similar to other studies (24). These perceived barriers underscore the importance of a systematic, bundled approach to care. Comprehensive implementation of the ABCDEF bundle, which pairs spontaneous awakening trials with spontaneous breathing trials, delirium assessment, and early mobility, is designed to mitigate these very concerns by promoting a culture of lighter sedation and proactive management.

The limitations of this study are as follows. First, this study employed convenience sampling, and participants were limited to ICU nurses from five tertiary general hospitals in Sichuan Province, China. Given the documented disparities in resources and protocols across different hospital tiers in China, the findings may not be representative of nurses in secondary or primary hospitals, or those in other provinces, which limits the generalizability of the conclusions. Second, the data were collected through self-report questionnaires, which may present a risk of reporting bias. Third, the analysis was limited to univariate comparisons. While the identified factors associated with knowledge and attitude scores, the lack of multivariate analyses means we could not control for confounders, thus limiting causal interpretation. Furthermore, the age distribution of our sample was skewed, with a majority of respondents being younger nurses. The potential for bias due to this demographic characteristic should be considered when interpreting the results. Future studies should adopt evaluation methods that are more objective. A large-sample, multi-center survey should be conducted nationwide to understand the cognition and practice of DSI among ICU nurses.

## Conclusion

In conclusion, this study identifies a critical gap between the high acceptance and the low implementation of Daily Sedation Interruption (DSI) among ICU nurses, primarily rooted in significant knowledge deficits and a lack of structured training. To bridge this gap, we recommend the development and implementation of evidence-based, simulation-enhanced DSI education programs and the formal integration of DSI protocols into nursing practice guidelines, with an emphasis on the “analgesia-first” principle. These targeted interventions are essential to translate positive attitudes into safe and routine practice, ultimately aiming to improve sedation management and patient outcomes in the ICU.

## Data Availability

The original contributions presented in the study are included in the article/supplementary material, further inquiries can be directed to the corresponding author.

## Footnotes

1
https://www.wjx.cn/
